# The Impact of Policy Guidelines on Hospital Antibiotic Use over a Decade: A Segmented Time Series Analysis

**DOI:** 10.1371/journal.pone.0092206

**Published:** 2014-03-19

**Authors:** Sujith J. Chandy, Girish S. Naik, Reni Charles, Visalakshi Jeyaseelan, Elena N. Naumova, Kurien Thomas, Cecilia Stalsby Lundborg

**Affiliations:** 1 Global Health, Department of Public Health Sciences, Karolinska Institutet, Stockholm, Sweden; 2 Department of Pharmacology & Clinical Pharmacology, Christian Medical College, Vellore, Tamil Nadu, India; 3 Department of Pharmacy, Christian Medical College, Vellore, Tamil Nadu, India; 4 Department of Biostatistics, Christian Medical College, Vellore, Tamil Nadu, India; 5 Department of Civil and Environmental Engineering, Tufts University School of Engineering, Medford, Massachusetts, United States of America; 6 Department of Gastrointestinal Sciences, Christian Medical College, Vellore, Tamil Nadu, India; 7 Department of Medicine, Christian Medical College, Vellore, Vellore, Tamil Nadu, India; Arizona State University, United States of America

## Abstract

**Introduction:**

Antibiotic pressure contributes to rising antibiotic resistance. Policy guidelines encourage rational prescribing behavior, but effectiveness in containing antibiotic use needs further assessment. This study therefore assessed the patterns of antibiotic use over a decade and analyzed the impact of different modes of guideline development and dissemination on inpatient antibiotic use.

**Methods:**

Antibiotic use was calculated monthly as defined daily doses (DDD) per 100 bed days for nine antibiotic groups and overall. This time series compared trends in antibiotic use in five adjacent time periods identified as ‘Segments,’ divided based on differing modes of guideline development and implementation: Segment 1– Baseline prior to antibiotic guidelines development; Segment 2– During preparation of guidelines and booklet dissemination; Segment 3– Dormant period with no guidelines dissemination; Segment 4– Booklet dissemination of revised guidelines; Segment 5– Booklet dissemination of revised guidelines with intranet access. Regression analysis adapted for segmented time series and adjusted for seasonality assessed changes in antibiotic use trend.

**Results:**

Overall antibiotic use increased at a monthly rate of 0.95 (SE = 0.18), 0.21 (SE = 0.08) and 0.31 (SE = 0.06) for Segments 1, 2 and 3, stabilized in Segment 4 (0.05; SE = 0.10) and declined in Segment 5 (−0.37; SE = 0.11). Segments 1, 2 and 4 exhibited seasonal fluctuations. Pairwise segmented regression adjusted for seasonality revealed a significant drop in monthly antibiotic use of 0.401 (SE = 0.089; *p*<0.001) for Segment 5 compared to Segment 4. Most antibiotic groups showed similar trends to overall use.

**Conclusion:**

Use of overall and specific antibiotic groups showed varied patterns and seasonal fluctuations. Containment of rising overall antibiotic use was possible during periods of active guideline dissemination. Wider access through intranet facilitated significant decline in use. Stakeholders and policy makers are urged to develop guidelines, ensure active dissemination and enable accessibility through computer networks to contain antibiotic use and decrease antibiotic pressure.

## Introduction

Infections remain a major cause of morbidity and mortality in the world especially in Low-and-Middle Income Countries (LMIC) [1], [2]. Antibiotics have been a significant force in reducing mortality in bacterial infections. However the number of effective antibiotics have been diminishing due to emerging bacterial resistance [3]. Recently identified mechanisms of bacterial resistance such as New Delhi metallo-β-lactamase 1 (NDM-1) may have grave consequences [4]. Resistance is not only rapidly spreading in hospitals in the cities, but also in the community and in rural areas [5]. The effectiveness of even life saving antibiotics have diminished with *Escherichia coli* resistance to ceftazidime and cefotaxime as high as 70% and 80% respectively [6] in intensive care settings.

Among the factors contributing to bacterial resistance, antibiotic consumption (hereafter referred to as antibiotic use), both individual and aggregate, appear to have a major role [7], [8]. Irrational use of antibiotics is a factor in many countries such as India [9]. In 2001, the WHO Strategy for Containment of Antimicrobial Resistance suggested steps to ensure rational use of antibiotics [10]. One of the major strategies suggested was formulation of antibiotic stewardship programmes with development of antibiotic policy guidelines being a core component [11]. The key purpose of guidelines is to improve rational antibiotic use. This activity has been well studied and more so in high income countries (HIC). A recent meta-analysis of 89 studies using clinical trials, interrupted time series and other methods assessed the effectiveness of antibiotic stewardship programs [12]. It showed that policy interventions changed antibiotic treatment and this was associated with significant improvement in outcomes. Unfortunately, there were hardly any studies from LMIC in this metanalysis. This is an important aspect to consider since infections and irrational antibiotic use are widely prevalent in these countries [9].

Another purpose of antibiotic stewardship is to contain antibiotic use. Containment is important as increased antibiotic use leading to environmental pressure contributes more to bacterial resistance [13]. Unfortunately, the outcome parameters for many of the studies in the metanalysis were mainly clinical and microbiological. Evidence is minimal especially in LMIC where regulatory environment, treatment practices and other factors may be vastly different to HIC.

The present study aims to bridge this gap and determine whether antibiotic policies are truly effective in LMIC. Computerized data on medicine use has not been widely available in many of these countries. It has been however available since 2002 in the institution where the analysis was conducted. This has facilitated assessment of antibiotic use trends and patterns over the last decade through this study. The institution has taken a leading role in India for development and dissemination of antibiotic guidelines. This study has therefore seized this opportunity to assess the impact of these guidelines and its role in containing antibiotic use in hospital inpatients over this ten year period. In addition, our study aims to compare different modes of guideline implementation and dissemination and see which mode is most effective in containment. This would be important in instituting effective and sustainable antibiotic policies, especially in LMIC where there are numerous challenges. These aspects of our study are unique and the findings could be crucial to health policy makers and hospital managements.

## Methods

### Study Setting

This study was carried out in a not-for-profit tertiary care teaching hospital in south India. This hospital caters to patients from various socioeconomic strata, from many parts of the country and has 2140 beds and more than 6000 outpatients per day [14]. The institution has a Drugs and Therapeutics Committee and its formulary subcommittee meets once a month. New antibiotics are introduced into the pharmacy only after peer review and discussion. The institution also has an Antibiotic Policy Committee, which is responsible for guidelines formulation and implementation.

A strict process is followed to evaluate pharmaceutical companies and their products before the medicine is stocked in the pharmacy. In addition, medicines are randomly analyzed for correct content using high performance liquid chromatography. Antibiotics in the hospital formulary are dispensed from various dispensing sections within the hospital after purchase with a prescription. Computer entry of the patient hospital number, medicine name, strength and number of units is made through the customized pharmacy software system.

### Antibiotic Policy of Hospital

There were three major phases of antibiotic policy guideline implementation during the study period i.e. between July 2002 and August 2012: one in 2005 (A), the second in 2009 (B), and the third in 2011 (C). Based on this, the study period was divided into the following segments:

Segment 1: July 2002 to February 2004 - This period was before the 2005 policy guidelines were introduced.

Segment 2: March 2004 to December 2005 - The Antibiotic Policy Committee with active participation of clinical departments and other stakeholders such as pharmacy and microbiology department initiated preparation for a comprehensive antibiotic policy in March 2004. The preparation phase included weekly meetings and active discussion of guidelines. Guidelines were finalized and widely distributed from January 2005 as a small booklet. Active dissemination lasted till December 2005. Therefore, Segment 2 was taken as a period which included preparation and dissemination from March 2004 to December 2005.

Segment 3: January 2006 to December 2008 - There was no active guideline dissemination during this period.

Segment 4: January 2009 to December 2010 - Guidelines were revised and published as a booklet in January 2009 and disseminated till end of 2010. Unlike guidelines disseminated and implemented in Segment 2, there was no sustained preparatory phase and discussion with all clinical departments. This segment was therefore taken from January 2009 to December 2010.

Segment 5: January 2011 to August 2012 - In January 2011, revised guidelines were published and distributed as a booklet and also made available through the intranet computer network of the hospital. Computers are available in every ward, outpatient room and departmental office of the hospital. This segment was therefore taken from January 2011 to August 2012.

### Data Collection and Antibiotic Use

Antibiotic use in inpatients was calculated using the hospital pharmacy computer system. Consumption was calculated as DDD (Defined Daily Doses) normalized for 100 bed days [15]. DDD per 100 bed days is an important indicator of inpatient antibiotic use and an objective measure of assessing changes in use due to interventions. Computer records of the dose and quantity of the antibiotic purchased allowed for calculation of number of DDDs. Inpatients do not receive antibiotics from sources outside the hospital during their hospital stay and hence all antibiotic use within the hospital was captured comprehensively. Bed days were calculated after receiving monthly hospitals admission data and the bed occupancy rate from medical records department.

Each antibiotic was calculated separately and coded as per DDD/ATC (Anatomical, Therapeutic and Chemical) Index [15]. Individual antibiotics were then categorized and DDD estimated for nine antibiotic groups: J01A - Tetracyclines, J01B - Amphenicols, J01C - Beta-lactam antibacterials, J01D - Other Beta-lactam antibacterials, J01E - Sulfonamides and trimethoprim, J01F - Macrolides and Lincosamides, J01G - Aminoglycosides, J01M - Quinolones and J01X - Other antibacterials. This data facilitated assessment of antibiotic group trends and patterns. Finally, the overall antibiotic DDD per 100 bed days for the entire antibiotic spectrum (J01) was calculated monthly. This formed the main basis of the time series from July 2002 to August 2012.

### Design and Analysis

Segmented time series design was used to assess impact of guidelines on antibiotic use across different segments. Each of the five segments in our study had a minimum of 20 monthly time points. Monthly DDD values were plotted using a standard time series plot and calendar plots to better depict monthly and seasonal variation in overall and specific antibiotic groups. The average values of DDD per hundred bed days were estimated for each segment and each antibiotic group. For exploratory purposes, we estimated the linear trend for each segment (Model 1) and examined the effect of seasonality on the segment trend for each outcome of interest (Model 2).

The models were formulated as:
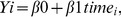
(Model 1)




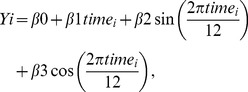
(Model 2)where 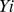 – are monthly values for overall DDD or DDD values in specific *i*-segments. This exploratory analysis was performed to characterize the trend (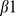) for each segment individually and correct for a potential seasonality effect (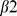 and 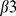) [16]–[18].

The exploratory analysis was conducted for each antibiotic group. For Models 1 and 2, the results were presented as values with corresponding confidence intervals (CI) for start and end dates for each segment.

In order to adjust for interruption implied by Models 1 and 2, we performed a pair-wise segmented regression adjusted for seasonality which also allows us to identify the changes in two consecutive segments [19].
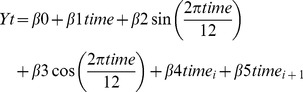
(Model 3)where 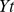 – are monthly values for overall DDD or DDD values for antibiotic groups in two adjacent *i*- and *i*+1-segments; *time_i_* – is a month sequence (in reverse order) for *i*-segment; *time_i_*_+1_ – is a month sequence for *i*+1-segment; *time* – is a month sequence for two adjacent segments to adjust for seasonality. The difference between regression parameters 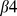 and 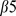 indicates changes in segment-specific trends. By assuming the expected normality of point estimates obtained via the OLS (Ordinary Least Squares) fitting procedure, the uncertainty measures for these regression parameters allow to directly compare trends in individual segments and to interpret the significance of standard z-test to infer the changes in adjacent segments [19]. For example, if one segment demonstrates no significant change while another segment shows a significant change, a difference between two segments can be inferred by proxy. Furthermore, non-overlapping 95% confidence intervals for 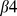 and 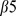 indicate high likelihood for a significant difference between two segments. For Model 3, the results were presented as predicted values for the start of the *i*-segment, start and end of the *i*+1-segment, and *p*-values indicating the standard z-test for a linear trend against H_0_: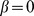, for *i*-segment and *i*+1-segments, respectively. For all models, the quality of fit was assessed by the R^2^ values. Model diagnostics showed no significant autocorrelation of residuals.

### Ethics Statement

Ethics permission was obtained from the Institutional Review Board of Christian Medical College Vellore, India (IRB (EC)-ER-4-10-03-2010). The analysis was done on aggregate data of antibiotic use from the pharmacy computer system of the institution. There was no direct human participation in this study and no attempt was made to identify individuals in the analysis. Informed consent was therefore waived.

## Results

Table 1 summarizes antibiotic use in specific segments. Individual antibiotic groups were arranged in descending order based on average monthly DDD/100 bed days at the beginning of the study period (Segment 1). In Segment 1, J01C and J01D were major contributors (29.0 and 27.6%), J01M and J01G were moderate contributors (17.1 and 12.1%) while J01X, J01E, J01A, J01F and J01B were minor contributors (5.9, 2.9, 2.6, 1.5, and 1.3%) to overall antibiotic use (J01).

**Table 1:** Descriptive summary of antibiotic use: average monthly DDD/100 bed days for individual segments with standard deviation (SD) for overall and nine individual antibiotic groups.

Antibiotic groups*	DDD/100 bed days (SD)				
Segments	1	2	3	4	5
J01	65.83 (6.67)	72.17 (3.15)	82.68 (4.63)	87.88 (4.37)	84.02 (3.90)
J01C	19.12 (2.33)	20.45 (1.47)	25.51 (2.63)	26.40 (1.68)	28.56 (1.79)
J01D	18.16 (2.12)	20.48 (1.19)	21.91 (1.72)	22.95 (0.91)	19.51 (2.18)
J01M	11.26 (1.58)	11.23 (1.03)	12.12 (1.11)	12.71 (1.00)	12.03 (1.13)
J01G	7.98 (0.75)	8.12 (0.61)	7.85 (0.64)	7.40 (0.48)	5.88 (0.46)
J01X	3.91 (0.44)	5.27 (0.75)	6.72 (0.75)	7.75 (0.72)	7.78 (0.70)
J01E	1.90 (0.27)	2.12 (0.28)	2.18 (0.56)	2.74 (0.49)	2.84 (0.45)
J01A	1.69 (0.43)	1.87 (0.46)	3.14 (1.00)	3.71 (1.24)	3.08 (1.13)
J01F	0.96 (0.41)	1.71 (0.44)	2.46 (0.66)	3.60 (0.80)	4.10 (0.66)
J01B	0.84 (0.17)	0.91(0.18)	0.78 (0.10)	0.62 (0.08)	0.54 (0.06)

*J01 - Overall, J01C - Beta-Lactam Antibacterials, J01D – Other Beta-Lactam Antibacterials, J01M - Quinolones, J01G-Aminoglycosides, J01X - Other Antibacterials, J01E - Sulfonamides and Trimethoprim, J01A - Tetracyclines, J01F - Macrolides and Lincosamides, J01B – Amphenicols.

**Figure 1:**
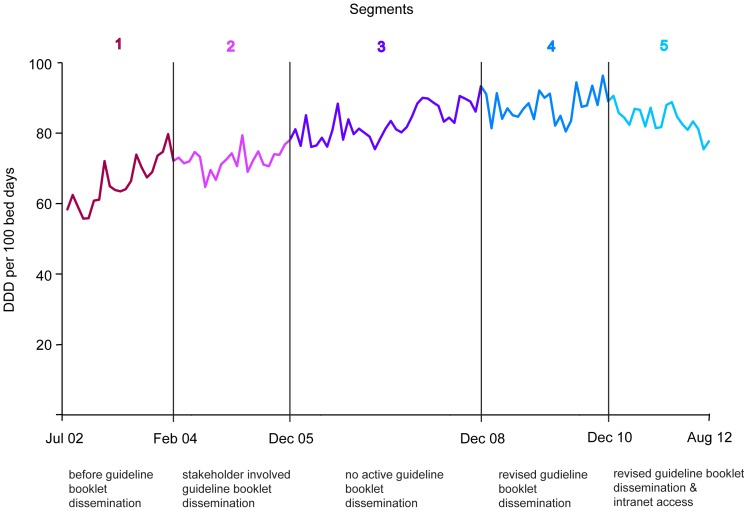
Time series of monthly antibiotic use over ten years with five segments demarcated by vertical lines.

The time series of monthly values for overall DDD/100 bed days are shown in Figure 1. Vertical lines separate individual segments and delineate general trend and potential seasonal oscillations. Antibiotic use showed a rising trend in Segments 1, 2 and 3, stabilized during Segment 4 and declined in Segment 5.

**Figure 2:**
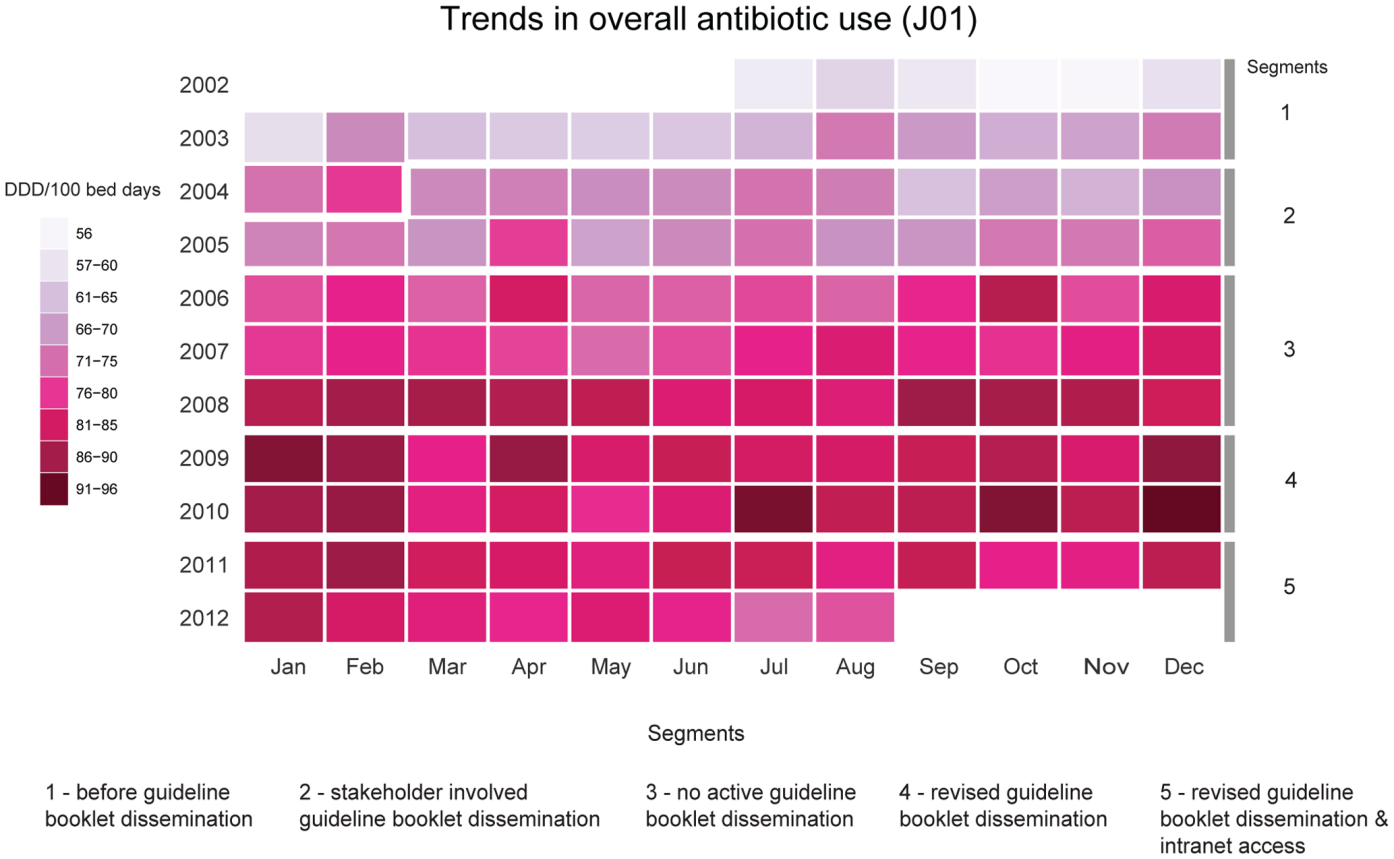
Calendar plot showing trend and seasonal patterns of overall antibiotic use.

**Figure 3:**
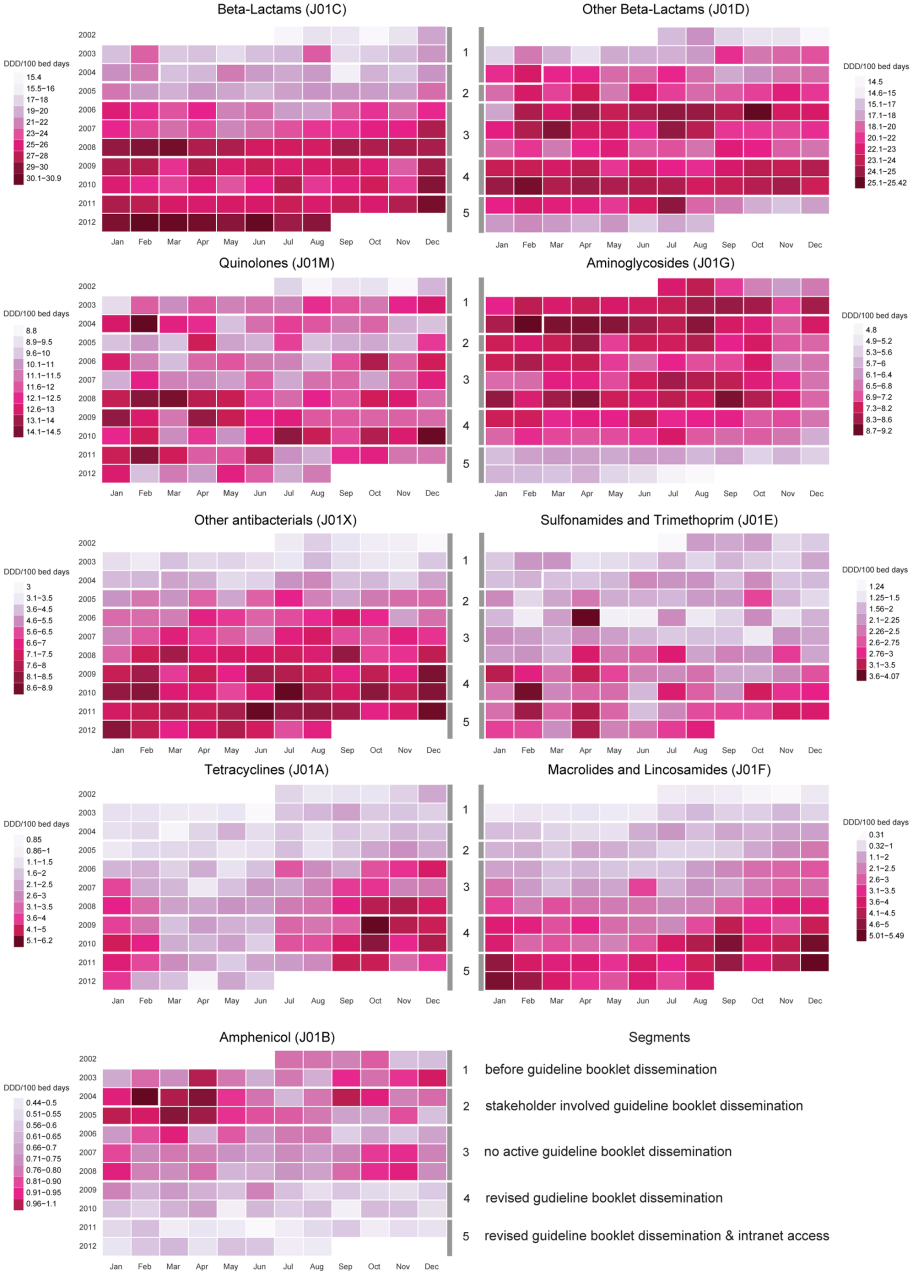
Seasonal patterns and trends in antibiotic use for nine groups.

To facilitate visualization of trends and seasonal patterns across antibiotic groups, a compact version of monthly DDD per 100 bed days for overall and individual antibiotic groups is shown using calendar plots (Figures 2 and 3). Seasonal variations with increased use during cooler months (October to February) and a spike in July 2010 in overall antibiotic group (J01) are apparent (Figure 2). Trends, seasonality, and spikes in individual antibiotic groups across the five segments over ten years shown in Figure 3 indicate the need to consider seasonal variations in the analysis.

To demonstrate the trend for overall antibiotic use with and without adjusting for seasonality, the results of the two regression models are presented in Table 2. The results include values for monthly rate (slope with standard error, *p*-values and quality of fit) and predicted values of DDD per 100 bed days for the start of the segment. The seasonality adjustment for overall antibiotic use was critical for Segments 1, 2, and 4. A significant increase of approximately 20 DDD per 100 bed days was observed in the first segment at a rate of 0.95 (SE = 0.18). Only Segment 5 showed a significant decrease in use at a rate of 0.37 (SE = 0.11) with a reduction of over 10 DDD per 100 bed days. Segment 3 however showed a significant rise in trend at a rate of 0.31 (p<0.001). As compared to Model 1, overall Model 2 improved quality of fit and better predicted DDD/100 bed days due to adjustment for seasonality (shown in bold letters).

**Table 2:** Exploratory analysis of trends in individual segments without (Model 1) and with (Model 2) adjustment for seasonality for overall antibiotic use (DDD/100 bed days).

Segment	Model 1		Model 2	
	Slope (SE), *p*-value*; R^2^ **	Predicted values with CI for start/end dates	Slope (SE), *p*-value*; R^2^ **	Predicted values with CI for start/end dates
1	0.97(0.16), *<0.001*; 0.63	56.68 (52.98–60.38) 75.16 (71.46–78.86)	0.95 (0.18), *<0.001*; 0.65	56.88 (52.67–61.08) 75.99 (71.79–80.20)
2	0.13(0.10), 0.211; 0.11	70.79 (68.37–73.21) 73.53 (71.11–75.95)	0.21 (0.08), *0.025;* 0.78	71.80 (69.57–74.03) 73.57 (71.34–75.81)
3	0.30 (0.07), *<0.001*; 0.49	77.44 (74.71–80.17) 87.90 (85.16–90.63)	0.31(0.06), *<0.001*; 0.49	79.04 (76.21–81.86) 89.60 (86.78–92.42)
4	0.10 (0.13), 0.418; 0.03	86.67 (83.35–89.98) 89.06 (85.74–92.37)	0.05 (0.10), 0.644, 0.61	89.33 (86.24–92.42) 91.13 (88.04–94.22)
5	−0.46 (0.14), *0.004*; 0.78	88.33 (85.26–91.40) 79.70 (76.60–82.74)	−0.37 (0.11), *0.004*, 0.59	89.68 (87.09–92.26) 78.86 (76.28–81.45)

*p-values below 0.05 are italicized

**R^2^- values that increase in the seasonally adjusted Model 2 are shown in bold

The results of this exploratory analysis for individual antibiotic groups are shown in Table S1. Seasonal variation in antibiotic use was apparent for all individual groups except J01B. J01M, J01G, J01X J01E, J01A and J01F exhibited significant seasonal fluctuations in use across all five segments, whereas J01C showed seasonality across the first four segments and J01D across the last four segments. A decline in monthly rate of antibiotic use in Segment 5 was noted in all groups except J01C.

To compare the trends in adjacent segments, a pair-wise segmented regression adjusted for seasonal variation (Model 3) is presented in Table 3. The modeling results confirm the observed significant increase in monthly DDD per 100 bed days in Segments 1 and 3 and a significant decline in Segment 5 resulting in the level decreasing to 79 DDD per 100 bed days similar to the beginning of Segment 3. There were no significant trends in Segments 2 and 4 (p = 0.926 and 0.629 respectively). Table S2 shows similar analysis for 9 antibiotic groups. J01D (p<0.001), J01M (p = 0.006), J01G (p<0.001) and J01A (p<0.001) showed significant decrease in monthly rate of antibiotic use in Segment 5, while the decline noted for J01X and J01F use was not significant. Only J01C showed a significant increase in antibiotic use (p<0.001).

**Table 3:** Pair-wise segmented analysis as the estimated rate of change in monthly DDD/100 bed days for adjacent segments and predicted values for the start of the *i*-segment, start and end of the *i*+1-segment.

S*	Slopes (SE) for two adjacent *i*-segment and *i*+1-segments	Predicted values for the start of the *i*-segment, start and end of the *i*+1-segment	R^2^	*p* value**
12	0.708 (0.120) 0.010 (0.107)	57.88, 74.23 and 73.01	0.61	*<0.001*0.926
23	0.265 (0.088) 0.362 (0.050)	71.32, 77.35 and 89.39	0.73	*0.004 <0.001*
3 4	0.273 (0.046) 0.036 (0.075)	79.70, 89.58 and 90.63	0.59	*<0.001*0.629
45	0.067 (0.072)–0.401(0.089)	89.15, 90.35 and 79.31	0.55	0.357 *<0.001*

*S – Segment.

**p values reflect the results of standard z-test for a linear trend for *i*-segment and *i*+1 segments, respectively; *p*-values below 0.05 are italicized.

## Discussion

The study looked at ten years of antibiotic use in a tertiary care hospital, which has patients coming from all over India and beyond. During this period, antibiotic guidelines were prepared and disseminated in various modes and each had its impact in their respective segments. Overall, there was a rising trend in antibiotic use except in Segments 4 and 5 during which stabilization and decline in trend was seen (Figure 1). There was seasonal increase in overall antibiotic use especially during the cooler months (Figure 2 and Table 2).

### Patterns, trends and seasonality of antibiotic groups

Individual antibiotic groups contributed to overall antibiotic use in different ways during various periods across the decade. Before discussing the impact of guidelines on overall use, the pattern of use in each antibiotic group is therefore highlighted below (Figure 3, and Tables S1 and S2).

Beta-lactam antibacterials (J01C): A rising trend was observed across all segments with maximum consumption in the last segment. This could be due to the increasing use of antibiotics such as piperacillin-tazobactam for multi-drug resistant hospital acquired infections, especially in critical care settings [6]. To tackle the problem of Extended Spectrum Beta Lactamase (ESBL), there has been decreased use of third and fourth generation cephalosporins for culture confirmed cases showing no ESBL. This may have contributed to increase in use of beta-lactams.

Other Beta-lactam antibacterials (J01D): The initial segments showed a rising trend that could be explained by increasing multidrug resistance. Carbapenems are the drugs of choice for treatment of ESBL infections [20]. However, cephalosporins and carbapenems were among the targeted antibiotic groups for containment in the policy introduced in Segment 5. This could explain the observed decline in Segment 5.

Quinolones (J01M): During the year, higher use was noticed in months of January and February. Quinolones are commonly prescribed empirically for respiratory infections including community acquired pneumonia and exacerbations of chronic obstructive pulmonary disease (COPD) [21], [22]. Quinolones are also commonly misused for “viral” respiratory infections which could explain observed seasonality. Their use in multidrug resistant TB is invaluable and is included for MDR TB regimens [23]. These reasons can cumulatively account for the high quinolone use noted in earlier segments. Quinolone use was targeted for containment as per policy changes introduced in Segment 5 and may explain the decline seen in this segment.

Aminoglycosides (J01G): This group showed a rapid decline in use after Segment 3. Availability of newer antibiotics with gram negative spectrum and serious side effects of aminoglycosides like nephrotoxicity and ototoxicity [24] may have contributed to its decline.

Other anti-bacterials (J01X): This group with glycopeptides and nitroimidazoles witnessed a rising trend in use. Nitroimidazoles to cover anaerobic infections and higher usage of glycopeptides to tackle rising incidence of Methicillin Resistant *Staphylococcus aureus* (MRSA) may have contributed to this trend [25].

Sulfonamides and trimethoprim (J01E): A spike in April 2006 with rising trend in Segments 4 and 5 could be explained by additional indications like HIV associated opportunistic infections and the rise of multi-drug resistant malaria [26], [27].

Tetracyclines (J01A): Seasonal fluctuation was observed in the calendar plot (Figure 3) and Model 2 (Table S1). Scrub typhus is endemic in the area of study and particularly seen in the rainy season and cooler months (October to February) [28]. Other rickettsial infections (undifferentiated acute febrile illness) could have also contributed. These responded well to doxycycline.

Amphenicols (J01B): A rapid decline in use was evident. This decline was predictable after the emergence of widespread resistance especially among salmonella species [29]. Safer and more efficacious alternatives could have contributed to this decline.

Macrolides and Lincosamides (J01F): An overall rising trend with seasonal fluctuations was apparent. Macrolides, especially azithromycin, are frequently prescribed for respiratory infections which flare up during the cold months of the year [21].

From Swedish hospitals, an increased use of penicillins with enzyme inhibitors (J01CR) such as piperacillin-tazobactam and carbapenems has been reported. Cephalosporins on the other hand, showed a rapid decline, especially the 2^nd^ generation cephalosporins [30]. The findings in our study have a similar trend, especially declining cephalosporins and increasing piperacillin-tazobactam. In both studies, carbapenem use increased till 2010 though subsequently it showed a decline in our study (Segment 5).

Among the few studies in India, an intensive care study showed that third generation cephalosporins and meropenem were frequently used [31]. Another study using the focus of infection approach to identify areas of improvement in antibiotic prescribing reported high rates of fluoroquinolone and third generation cephalosporin prescriptions [32]. Our findings mirror antibiotic patterns in these studies but with the added advantage of observing antibiotic use over ten years.

### Impact of booklet guideline implementation on overall antibiotic use

The rising trend of antibiotic use seen in Segment 1 changed as guidelines were introduced at various periods across the decade. In Segment 2, a decline in trend in antibiotic use was seen though not statistically significant (Table 2 and 3). This period was characterized by intense stakeholder participation for guideline preparation. This kind of participatory activity would have spurred clinical departments to comply more with the guidelines and may have led to a decline in slope in Segment 2. Segment 3 had no active dissemination of guidelines and this could explain the marked increase in trend. This finding stresses the need for continuous exposure to guidelines and reinforcement. Updating new changes during this period was difficult due to the intense participatory nature of preparations in the earlier period, consequently inhibiting long term sustainability. A revised guidelines booklet was disseminated in Segment 4, during which there was no change in trend of antibiotic use. This shows that guideline dissemination periods may help to contain but not reduce antibiotic use. In addition, to physically carry a book in every instance and refer to guidelines may not be practical on a continuous basis.

### Impact of online intranet access to policy guidelines on overall antibiotic use

Segment 5 was marked by dissemination of policy guidelines through a booklet and intranet access in all outpatient, departmental offices and ward computers. During this period, a significant decrease in antibiotic use was seen (Table 2 and 3). Intranet policy guideline strategy is possible in centers where there is widespread access to computers in wards, outpatient departments (OPDs), intensive care units (ICUs) and other critical areas of hospitals. Our hospital has such facilities making such a strategy feasible. Where such facilities are available, this mode of policy implementation has the advantage of widespread and instant dissemination of standard guidelines with possibility of frequent updates. It can be reviewed by doctors and all healthcare professionals anytime within the hospital. Instantaneous feedback and query through email is also possible which facilitates an interactive dynamism between the user and other stakeholders.

In LMICs, the number of hospitals with hospital information system (HIS) is growing. However many do not have antibiotic policy guidelines let alone accessibility through the computer network. This strategy however should be explored by hospitals which have such capacity. Centers that can incorporate a similar mode of guideline dissemination may have a more effective strategy in containing antibiotic use as evidenced through this study. Consequently, resistance burden may reduce, ultimately leading to better healthcare and cost related benefits. A lack of infrastructure in some government facilities may hinder this strategy. Bigger government hospitals may have computer networks and therefore the situation is hopeful. Another encouraging situation is that more and more LMIC countries have fast developing information technology (IT) networks. This potential should be used by health policy makers and governments to their advantage for effective antibiotic guideline dissemination.

The model of computer network application has been demonstrated in developed countries. A university hospital in Sweden implemented persuasive antibiotic policy guidelines in the wake of an ESBL producing *Klebsiella pneumoniae* outbreak [33]. The policy was made available on the local intranet. The study used Interrupted Time Series (ITS) design to demonstrate significant containment in prescription of 3^rd^ and 4^th^ generation cephalosporins and prevent increase in fluoroquinolone and carbapenem prescriptions. An intensive care unit (ICU) based prospective study done in Australia showed how implementing a computerized decision support tool resulted in 10.5% reduction of overall antibiotic consumption (166 to 149 DDDs/100 ICU bed days) [34]. Similarly, other studies done in HIC have demonstrated decrease in antibiotic use through appropriate strategies for disseminating educational interventions [12], [35], [36]. Our study proves the developing nature of the country is no bar to this approach, provided IT capacity is developed.

### Limitations of the study

The study did not assess rationality of antibiotic prescriptions and adherence of physicians to the policy guidelines. The observed trends could have been influenced by other factors due to changes in the hospital over the decade. These factors include bed capacity, number of doctors, introduction of new laboratory tests and automation, antibiotic use audits and role of other health professionals such as clinical pharmacists. However, many of these factors have played a role throughout this 10 year period and thereby their impact on decreasing antibiotic use in Segment 5 alone would be minimal. Linking antibiotic use data with resistance rates and clinical outcome such as mortality rates would have given additional information which is being planned for a future study.

## Conclusion

Overall, the rising trend in antibiotic use was contained towards the latter half of the decade. Most antibiotic groups followed this trend. Varied patterns and seasonal fluctuations were observed. The development of antibiotic policy guidelines and its dissemination contributed significantly to containing antibiotic use. The study also showed that mode of policy implementation was critical to the effectiveness of policies as formulation of appropriate policies itself. The period where guidelines were also disseminated through intranet access showed a significant reduction in antibiotic use. Where facilities are available therefore, in addition to guideline booklet dissemination by standard methods, computer networking should be used effectively to broaden the accessibility of the antibiotic policy to a much wider group of physicians and healthcare personnel. Using this type of channel will increase the flexibility for frequent updating of information, will provide additional value to physicians and a dynamic platform for effective use of antibiotics. The findings of this study would encourage hospitals in LMICs to develop and implement antibiotic policy guidelines and use modern technology for wider stakeholder access. These measures may help contain antibiotic use and thereby decrease antibiotic pressure.

## Supporting Information

Table S1 **Exploratory analysis of trends in individual segments without (Model 1) and with (Model 2) adjustment for seasonality in nine antibiotic groups.**
(DOCX)

Table S2 **Pair-wise segmented analysis (for individual antibiotic groups) as the estimated rate of change in monthly DDD values for adjacent segments and predicted values for the beginning of the** ***i*** **-segment, beginning and end of the** ***i*** **+1-segment.**
(DOCX)
