# Correction: The Impact of Policy Guidelines on Hospital Antibiotic Use over a Decade: A Segmented Time Series Analysis

**DOI:** 10.1371/journal.pone.0102193

**Published:** 2014-07-01

**Authors:** The PLOS ONE Staff

There is an error in Table 3. In the first column, there is a spacing error that resulted in merged rows. Please see the corrected Table 3 here.

**Table 3:** Pair-wise segmented analysis as the estimated rate of change in monthly DDD/100 bed days for adjacent segments and predicted values for the start of the *i*-segment, start and end of the *i*+1-segment.

S*	Slopes (SE) for two adjacent *i*-segment and *i*+1-segments	Predicted values for the start of the *i*-segment, start and end of the *i*+1-segment	R^2^	*p* value**
12	0.708 (0.120)0.010 (0.107)	57.88, 74.23 and 73.01	0.61	*<0.001*0.926
23	0.265 (0.088)0.362 (0.050)	71.32, 77.35 and 89.39	0.73	*0.004* *<0.001*
34	0.362 (0.050)0.036 (0.075)	79.70, 89.58 and 90.63	0.59	*<0.001*0.629
45	0.067 (0.072)-0.401(0.089)	89.15, 90.35 and 79.31	0.55	0.357*<0.001*

^*^S - Segment

^**^ *p* values reflect the results of standard z-test for a linear trend for *i*-segment and *i*+1 segments, respectively; *p*-values below 0.05 are italicized.
