# Supplementary material for: The Impact of Policy Guidelines on Hospital Antibiotic Use over a Decade: A Segmented Time Series Analysis
Source: PLoS One. 2014 Mar 19;9(3):e92206. doi: 10.1371/journal.pone.0092206 (PMC3960230; doi:10.1371/journal.pone.0092206)
Supplement: Table S1 — Exploratory analysis of trends in individual segments without (Model 1) and with (Model 2) adjustment for seasonality in nine antibiotic groups. (DOCX) [file pone.0092206.s001.docx]

**Table S1.** Exploratory analysis for trends of different segments for each antibiotic group.

| **Segment** | **Model 1** | | **Model 2** | |
| --- | --- | --- | --- | --- |
|  | **Slope (se), *p*-value*; R^2^** | **Predicted values with CI for start/end dates** | **Slope (se), *p*-value; R^2**^** | **Predicted values with CI for start/end dates** |
|  | **J01C-Beta Lactams** | | | |
| 1 | 0.24 (0.08), *0.007*; 0.30 | 16.88 (15.19 - 18.58) 21.35 (19.65 - 23.05) | 0.19 (0.07), *0.012*; **0.59** | 16.54 (14.91 - 18.18) 22.11 (20.48 - 23.74) |
| 2 | 0.08 (0.05),  0.147; 0.05 | 19.65 (18.43 - 20.87)  21.26 (20.04 - 22.48) | 0.09 (0.05), 0.096; **0.19** | 20.12 (18.75 - 21.49) 21.50 (20.13 - 22.87) |
| 3 | 0.15 (0.05),  *0.008*; 0.66 | 22.98 (20.74 - 25.22)  28.37 (26.13 - 30.60) | 0.18 (0.04),  *< 0.001*; **0.74** | 24.06 (22.37 - 25.74) 29.90 (28.21 - 31.59) |
| 4 | -0.06 (0.04),  0.127; 0.64 | 26.98 (26.60 - 27.91)  25.69 (24.77 - 26.62) | -0.07 (0.04),  0.062; **0.75** | 27.29 (26.16 - 28.41) 25.79 (24.67 - 26.92) |
| 5 | 0.17 (0.08),  *0.042*; 0.86 | 26.98 (25.19 - 28.77)  30.27 (28.48 - 32.06) | 0.27 (0.03),  *< 0.001*; 0.86 | 27.17 (26.45 - 27.90) 29.77 (29.05 - 30.50) |
| **J01D – Other beta-lactams** | | | | |
| 1 | 0.27 (0.07), *0.001;* 0.56 | 15.64 (14.04 - 17.24)  20.83 (19.23 - 22.44) | 0.28 (0.08),  *0.003;* 0.56 | 15.77 (13.89 - 17.65) 20.76 (18.87 - 22.64) |
| 2 | 0.04 (0.05), 0.374; 0.35 | 20.06 (18.96 - 21.16)  20.93 (19.83 - 22.02) | 0.09 (0.03),  *0.004;* **0.79** | 20.58 (19.88 - 21.29) 21.15 (20.44 - 21.85) |
| 3 | -0.09 (0.03), *0.002;* 0.16 | 23.42 (22.41 - 24.44)  20.36 (19.35 - 21.38) | -0.09 (0.02),  *<0.001;* **0.40** | 22.80 (21.72 - 23.88) 19.63 (18.54 - 20.71) |
| 4 | 0.05 (0.04), 0.201; 0.82 | 22.40 (21.45 - 23.36)  23.48 (22.53 - 24.44) | 0.08 (0.02),  *<0.001;* 0.80 | 22.71 (22.17 - 23.25) 24.32 (23.78 - 24.86) |
| 5 | -0.28 (0.07), *0.001;* 0.75 | 22.20 (20.62 - 23.77)  16.80 (15.23 - 18.37) | -0.32 (0.05),  *<0.001;* **0.78** | 21.51(20.37 - 22.65) 16.69 (15.54 - 17.83) |
| **J01M-Quinolones** | | | | |
| 1 | 0.24 (0.02), *<0.001*; 0.87 | 8.95 (8.44 - 9.46)  13.55 (13.04 - 14.06) | 0.23 (0.01), *< 0.001*; **0.97** | 9.03 (8.69 - 9.38) 13.84 (13.49 - 14.19) |
| 2 | -0.05 (0.03), 0.108; 0.03 | 11.74 (11.03 - 12.44)  10.70 (10.00 - 11.40) | -0.04 (0.03),  0.214; **0.24** | 11.63 (10.84 - 12.42)  10.45 (9.66 - 11.25) |
| 3 | 0.04 (0.02), 0.113; 0.34 | 11.50 (10.62 - 12.38)  12.75 (11.87 - 13.63) | 0.04 (0.02),  *0.037;* 0.34 | 11.89 (10.98 - 12.80)  13.24 (12.33 - 14.16) |
| 4 | 0.02 (0.03), 0.656; 0.29 | 12.56 (11.65 - 13.47)  12.92 (12.01 - 13.83) | 0.02 (0.04),  0.685; **0.32** | 12.70 (11.52 - 13.88)  13.07 (11.89 - 14.25) |
| 5 | -0.11 (0.03), *0.007*; 0.29 | 13.04 (12.28 - 13.80)  11.02 (10.26 - 11.78) | -0.10 (0.04),  *0.017*; **0.32** | 13.15 (12.26 - 14.04)  10.87 (9.98 - 11.76) |
| **J01G-Aminoglycosides** | | | | |
| 1 | 0.08 (0.03), *0.033*; 0.60 | 7.29 (6.55 - 8.04)  8.76 (8.02 - 9.50) | 0.09 (0.03), *0.007*; **0.64** | 7.58 (6.92 - 8.24) 8.80 (8.14 - 9.46) |
| 2 | -0.06 (0.02) *0.004;* 0.65 | 8.79 (8.32 - 9.26)  7.45 (6.98 - 7.93) | -0.04 (0.01), *0.004;* 0.63 | 8.72 (8.37 - 9.06) 7.30 (6.94 - 7.64) |
| 3 | 0.01 (0.01),  0.407; 0.44 | 7.62 (7.02 - 8.22) 8.06 (7.46 - 8.66) | 0.02 (0.01),  0.267; 0.42 | 7.38 (6.74 - 8.02) 7.88 (7.24 - 8.52) |
| 4 | -0.05 (0.01),  *0.001*; 0.76 | 8.00 (7.64 - 8.35)  6.78 (6.43 - 7.14) | -0.05 (0.01),  *<0.001*; 0.64 | 7.71 (7.42 - 8.00) 6.61 (6.32 - 6.91) |
| 5 | -0.06(0.01), *0.001*; 0.63 | 6.42 (6.10 - 6.74)  5.31 (4.99 - 5.63) | -0.06 (0.02),  *0.002*; **0.68** | 6.37 (6.01 - 6.74)  5.21 (4.85 - 5.57) |
| **J01X-Other antibacterials** | | | | |
| 1 | 0.04 (0.02),  *0.033*; 0.21 | 3.54 (3.16 to 3.91) 4.29 (3.91 to 4.67) | 0.04 (0.01),  *0.015*; **0.44** | 3.67 (3.32 to 4.02) 4.36 (4.01 to 4.71) |
| 2 | 0.07 (0.02),  *0.003*; 0.36 | 4.51 (3.98 to 5.03)  6.04 (5.51 to 6.56) | 0.09 (0.02),  *0.001*; **0.50** | 4.56 (4.01 to 5.10)  5.97 (5.42 to 6.51) |
| 3 | 0.04 (0.01), *0.001*; 0.26 | 6.00 (5.54 to 6.45)  7.45 (7.00 to 7.91) | 0.04 (0.01),  *<0.001*; **0.36** | 5.75 (5.25 to 6.25)  7.21 (6.71 to 7.71) |
| 4 | 0.02 (0.02),  0.243; 0.06 | 7.46 (6.92 to 8.01)  8.03 (7.49 to 8.58) | 0.02 (0.02),  0.417; **0.22** | 7.75 (7.13 to 8.34)  8.24 (7.62 to 8.86) |
| 5 | -0.04 (0.03),  0.207; 0.33 | 8.17 (7.43 to 8.91)  7.34 (6.60 to 8.07) | -0.05 (0.04),  0.234; **0.34** | 8.10 (7.23 to 8.98)  7.35 (6.48 to 8.22) |
| **J01E-Sulfonamides and Trimethoprim** | | | | |
| 1 | 0.001 (0.010),  0.949; 0.14 | 1.89 (1.67 - 2.12)  1.91 (1.68 - 2.13) | -0.003 (0.010),  0.816; **0.27** | 1.82 (1.57 - 2.08)  1.92 (1.67 - 2.18) |
| 2 | 0.005 (0.009),  0.562; 0.08 | 2.0618 (1.84 - 2.28)  2.17 (1.96 - 2.39) | 0.005 (0.010),  0.596; **0.29** | 1.95 (1.71 - 2.18)  2.07 (1.84 - 2.31) |
| 3 | 0.018 (0.008),  *0.035*; 0.13 | 1.88 (1.56 - 2.19)  2.49 (2.17 - 2.81) | 0.023 (0.007),  *0.003*; **0.32** | 1.83 (1.50 - 2.16)  2.48 (2.15 - 2.81) |
| 4 | -0.003 (0.015),  0.867; 0.02 | 2.77 (2.37 - 3.17)  2.7112 (2.31 - 3.11) | 0.007 (0.013),  0.574; **0.19** | 2.94 (2.56 - 3.32)  2.99 (2.61 - 3.37) |
| 5 | -0.002 (0.017),  0.926; 0.06 | 2.86 (2.50 - 3.22)  2.83 (2.47 - 3.19) | 0.004 (0.004),  0.759; **0.43** | 3.07 (2.73 - 3.41)  2.75 (2.41 - 3.09) |
| **J01A-Tetracyclines** | | | | |
| 1 | 0.01 (0.02),  0.518; 0.09 | 1.56(1.08 - 2.05)  1.84(1.35 - 2.32) | 0.02 (0.02),  0.204; **0.37** | 1.44(1.80 - 1.81)  1.66 (1.29 - 2.02) |
| 2 | 0.04 (0.02),  0.073; 0.18 | 1.50 (1.05 - 1.95)  2.24 (1.79 - 2.69) | 0.02 (0.02),  0.242; **0.21** | 1.47 (1.04 - 1.91)  2.26 (1.82 - 2.70) |
| 3 | 0.05 (0.03),  *0.042*; 0.10 | 2.22 (1.16 - 3.28)  4.14 (3.08 - 5.20) | 0.04 (0.01),  *0.002*; **0.64** | 2.84 (2.33 - 3.36)  4.53 (4.01 - 5.04) |
| 4 | 0.06 (0.06),  0.331; 0.14 | 3.13 (1.54 – 4.72)  4.47 (2.88 – 6.06) | 0.01 (0.01),  0.411; **0.89** | 4.22 (3.81 – 4.63)  5.08 (4.67 – 5.49) |
| 5 | -0.10 (0.08),  0.208; 0.31 | 3.97 (2.36 - 5.57)  2.21 (0.60 - 3.82) | -0.06 (0.02),  *0.009*; **0.84** | 4.30 (3.81 - 4.79)  1.76 (1.27 - 2.25) |
| **J01F-Macrolides and Lincosamides** | | | | |
| 1 | 0.06 (0.01),  *<0.001*; 0.62 | 0.42 (0.20 - 0.65)  1.51 (1.28 - 1.73) | 0.06 (0.01),  *<0.001*; **0.72** | 0.46 (0.23 - 0.69)  1.43 (1.20 - 1.66) |
| 2 | 0.04 (0.02),  0.140; 0.05 | 1.34 (0.74 - 1.95)  2.13 (1.52 - 2.73) | 0.02 (0.02),  0.321; **0.15** | 1.47 (0.94 - 2.00)  2.21 (1.68 - 2.75) |
| 3 | 0.04 (0.01),  *0.001*; 0.29 | 1.71 (1.25 - 2.17)  3.23 (2.77 - 3.69) | 0.04 (0.01),  <*0.001;* **0.66** | 2.11 (1.79 - 2.42)  3.59 (3.27 - 3.90) |
| 4 | 0.06 (0.03),  0.112; 0.27 | 3.04 (2.15 - 3.94)  4.31 (3.41 - 5.21) | 0.03 (0.02),  0.146; **0.49** | 3.53 (2.85 - 4.21)  4.58 (3.90 - 5.26) |
| 5 | -0.03 (0.04),  0.486; 0.45 | 4.40 (3.50 - 5.31)  3.86 (2.95 - 4.77) | -0.002 (0.02),  0.894; **0.64** | 4.80 (4.38 - 5.22)  3.94 (3.51 - 4.36) |
| **J01B-Amphenicols** | | | | |
| 1 | 0.016 (0.006),  *0.023*; 0.27 | 0.70 (0.56 - 0.83)  0.99 (0.86- 1.13) | 0.014 (0.007),  0.051; **0.31** | 0.70 (0.54 - 0.86)  1.03 (0.87 - 1.19) |
| 2 | -0.014 (0.009),  0.127; 0.40 | 1.05 (0.84 - 1.27)  0.76 (0.55 - 0.98) | -0.007 (0.006),  0.238; **0.47** | 1.13 (0.98 - 1.29)  0.85 (0.70 - 1.01) |
| 3 | 0.001 (0.002),  0.540; 0.02 | 0.76 (0.68 - 0.84)  0.81 (0.72 - 0.89) | 0.001 (0.002),  0.428; **0.12** | 0.81 (0.73 - 0.89)  0.86 (0.77 - 0.94) |
| 4 | -0.005 (0.002),  *0.007*; 0.16 | 0.68 (0.63 - 0.73)  0.55 (0.51 - 0.60) | -0.004 (0.002),  *0.039*; **0.37** | 0.67 (0.62 - 0.72)  0.57 (0.51 - 0.62) |
| 5 | 0.001 (0.003),  0.665; 0.18 | 0.53 (0.47 - 0.59)  0.55 (0.49 - 0.61) | 0.003 (0.002), 0.233; **0.22** | 0.54 (0.49 - 0.60)  0.53 (0.47 - 0.59) |

* p values less than 0.05 are italicized

** R^2^ - values that increase in the seasonally adjusted Model 2 are shown in bold
