# Supplementary material for: The Impact of Policy Guidelines on Hospital Antibiotic Use over a Decade: A Segmented Time Series Analysis
Source: PLoS One. 2014 Mar 19;9(3):e92206. doi: 10.1371/journal.pone.0092206 (PMC3960230; doi:10.1371/journal.pone.0092206)
Supplement: Table S2 — Pair-wise segmented analysis (for individual antibiotic groups) as the estimated rate of change in monthly DDD values for adjacent segments and predicted values for the beginning of the i -segment, beginning and end of the i +1-segment. (DOCX) [file pone.0092206.s002.docx]

**Table S2.** The results of pair-wise segmented analysis (for individual antibiotic groups) as the estimated rate of change in monthly DDD values for adjacent segments and predicted values for the beginning of the *i*-segment, beginning and end of the *i*+1-segment.

| **S^*^** | **Slopes (SE) for two adjacent *i*-segment and *i*+1-segments** | **Predicted values for the beginning of the *i*-segment, beginning and end of the *i*+1-segment,** | **R^2^** | ***p*-value^**^** |
| --- | --- | --- | --- | --- |
| **J01C-Beta-lactams** | | | | |
| 12 | 0.134 (0.050) 0.031 (0.044) | 16.96, 21.16 and 21.42 | 0.43 | *0.010* 0.490 |
| 23 | 0.110 (0.050) 0.196 (0.028) | 20.38, 23.15 and 29.64 | 0.69 | *0.032 <0.001* |
| 34 | 0.145 (0.031) -0.100(0.050) | 23.91, 29.02 and 26.63 | 0.47 | *<0.001*0.050 |
| 45 | -0.037 (0.034)  0.251 (0.042) | 27.56, 26.92 and 13.32 | 0.56 | 0.280 *<0.001* |
| **J01D - Other beta-lactams** | | | | |
| 12 | 0.214 (0.052)  0.025 (0.047) | 15.95, 20.79 and 20.79 | 0.50 | *<0.001* 0.602 |
| 23 | 0.155 (0.045)  -0.049 (0.025) | 19.48, 22.19 and 20.25 | 0.34 | *0.001* 0.059 |
| 34 | -0.050 (0.023)  0.127 (0.036) | 22.72, 21.04 and 23.84 | 0.04 | *0.030 0.001* |
| 45 | 0.030 (0.038)  -0.338 (0.047) | 22.41, 22.78 and 16.32 | 0.78 | 0.442 *<0.001* |
| **J01M-Quinolones** | | | | |
| 12 | 0.155 (0.035)  -0.102 (0.031) | 9.47, 12.82 and 10.39 | 0.39 | *<0.0010.002* |
| 23 | -0.022 (0.029)  0.053 (0.016) | 11.90, 11.41 and 13.11 | 0.28 | 0.454 *0.002* |
| 34 | 0.034 (0.017)  0.007 (0.027) | 11.85, 13.08 and 13.17 | 0.25 | *0.045* 0.792 |
| 45 | 0.021 (0.026)  -0.094 (0.032) | 12.64, 13.06 and 10.97 | 0.27 | 0.410 *0.006* |
| **J01G-Aminoglycosides** | | | | |
| 12 | 0.073 (0.017)  -0.055 (0.015) | 7.58, 8.84 and 7.19 | 0.54 | *<0.0010.001* |
| 23 | -0.053 (0.016)  0.019 (0.009) | 8.75, 7.31 and 7.89 | 0.44 | *0.002 0.042* |
| 34 | 0.015 (0.009)  -0.055 (0.014) | 7.38, 7.85 and 6.55 | 0.45 | 0.105 *<0.001* |
| 45 | -0.055 (0.010)  -0.076 (0.013) | 7.88, 6.48 and 5.22 | 0.87 | *<0.001<0.001* |
| **J01X-Other antibacterials** | | | | |
| 12 | 0.036 (0.015)  0.083 (0.013) | 3.69, 4.58 and 5.95 | 0.76 | *0.019 <0.001* |
| 23 | 0.071 (0.015)  0.039 (0.009) | 4.54, 5.89 and 7.20 | 0.67 | *<0.001<0.001* |
| 34 | 0.040 (0.010) 0.023 (0.015) | 5.96, 7.44 and 7.98 | 0.44 | *<0.001*0.137 |
| 45 | 0.028 (0.018) -0.035 (0.023) | 7.55, 8.19 and 7.41 | 0.12 | 0.129 0.134 |
| **J01E-Sulfonamides and Trimethoprim** | | | | |
| 12 | 0.008 (0.009)  0.009 (0.008) | 1.87, 1.96 and 2.18 | 0.20 | 0.345 0.257 |
| 23 | -0.007 (0.011)  0.013 (0.006) | 2.21, 1.96 and 2.37 | 0.12 | 0.512 *0.034* |
| 34 | 0.023 (0.006)  0.010 (0.009) | 1.93, 2.76 and 2.85 | 0.46 | *<0.001*0.289 |
| 45 | 0.003 (0.009)  0.008 (0.011) | 2.96, 3.03 and 2.68 | 0.18 | 0.763 0.501 |
| **J01A-Tetracyclines** | | | | |
| 12 | 0.012 (0.013)  0.012 (0.011) | 1.47, 1.52 and 2.21 | 0.32 | 0.358 0.318 |
| 23 | 0.023 (0.020)  0.049 (0.011) | 1.30, 2.51 and 4.47 | 0.56 | 0.237 *<0.001* |
| 34 | 0.028 (0.008)  0.010 (0.014) | 3.08, 4.10 and 4.81 | 0.72 | *0.002* 0.479 |
| 45 | 0.015 (0.011)  -0.062 (0.015) | 4.11, 4.40 and 1.64 | 0.88 | 0.178 *<0.001* |
| **J01F- Macrolides and Lincosamides** | | | | |
| 12 | 0.051 (0.015)  0.022 (0.013) | 0.38, 1.45 and 2.09 | 0.47 | *0.002* 0.118 |
| 23 | 0.005 (0.011)  0.038 (0.006) | 1.59, 2.10 and 3.52 | 0.64 | 0.665 *<0.001* |
| 34 | 0.036 (0.008)  0.040 (0.013) | 2.14, 3.49 and 4.56 | 0.67 | *<0.0010.002* |
| 45 | 0.043 (0.016)  0.001 (0.019) | 3.57, 4.60 and 4.06 | 0.51 | *0.009* 0.976 |
| **J01B- Amphenicols** | | | | |
| 12 | 0.013 (0.005) -0.008 (0.005) | 0.69, 1.08 and 0.83 | 0.30 | *0.020* 0.127 |
| 23 | -0.012 (0.004) 0.001 (0.002) | 1.10, 0.83 and 0.85 | 0.37 | *0.002* 0.589 |
| 34 | -0.001 (0.002) -0.010 (0.002) | 0.81, 0.78 and 0.55 | 0.39 | 0.691 *<0.001* |
| 45 | -0.006 (0.002) 0.001 (0.002) | 0.70, 0.56 and 0.52 | 0.41 | *<0.001*0.760 |

^*^S - Segment

^**^ *p* values less than 0.05 are italicized
